# Differential Developmental Deficits in Retinal Function in the Absence of either Protein Tyrosine Sulfotransferase-1 or -2

**DOI:** 10.1371/journal.pone.0039702

**Published:** 2012-06-22

**Authors:** David M. Sherry, Yogita Kanan, Robert Hamilton, Adam Hoffhines, Kelsey L. Arbogast, Steven J. Fliesler, Muna I. Naash, Kevin L. Moore, Muayyad R. Al-Ubaidi

**Affiliations:** 1 Department of Cell Biology, University of Oklahoma Health Sciences Center, Oklahoma City, Oklahoma, United States of America; 2 Oklahoma Center for Neurosciences, University of Oklahoma Health Sciences Center, Oklahoma City, Oklahoma, United States of America; 3 Department of Pharmaceutical Sciences, University of Oklahoma Health Sciences Center, Oklahoma City, Oklahoma, United States of America; 4 Research Service, VA Western New York Healthcare System, University at Buffalo/State University of New York, Buffalo, New York, United States of America; 5 Departments of Ophthalmology and Biochemistry, University at Buffalo/State University of New York, Buffalo, New York, United States of America; 6 The SUNY Eye Institute, Buffalo, New York, United States of America; 7 Cardiovascular Biology Research Program, Oklahoma Medical Research Foundation, Oklahoma City, Oklahoma, United States of America; 8 Departments of Cell Biology and Medicine, University of Oklahoma Health Sciences Center, Oklahoma City, Oklahoma, United States of America; University of Florida, United States of America

## Abstract

To investigate the role(s) of protein-tyrosine sulfation in the retina and to determine the differential role(s) of tyrosylprotein sulfotransferases (TPST) 1 and 2 in vision, retinal function and structure were examined in mice lacking TPST-1 or TPST-2. Despite the normal histologic retinal appearance in both *Tpst1^−/−^* and *Tpst2^−/−^* mice, retinal function was compromised during early development. However, *Tpst1^−/−^* retinas became electrophysiologically normal by postnatal day 90 while *Tpst2^−/−^* mice did not functionally normalize with age. Ultrastructurally, the absence of TPST-1 or TPST-2 caused minor reductions in neuronal plexus. These results demonstrate the functional importance of protein-tyrosine sulfation for proper development of the retina and suggest that the different phenotypes resulting from elimination of either TPST-1 or -2 may reflect differential expression patterns or levels of the enzymes. Furthermore, single knock-out mice of either TPST-1 or -2 did not phenocopy mice with double-knockout of both TPSTs, suggesting that the functions of the TPSTs are at least partially redundant, which points to the functional importance of these enzymes in the retina.

## Introduction

Protein-tyrosine sulfation is one of many post-translational modifications that proteins can undergo in the cell. Although protein-tyrosine sulfation is utilized from plants to animals [1], our understanding of the functional role of protein-tyrosine sulfation is still in its infancy. Protein-tyrosine sulfation is performed in the *trans* Golgi network [2] by one of two independent tyrosylprotein sulfotransferases (TPST, 3′-phosphoadenylyl-sulfate:protein-tyrosine *O*-sulfotransferase, EC 2.2.8.20), TPST-1 and TPST-2 [3]. The TPSTs catalyze protein-tyrosine sulfation through the covalent transfer of a sulfate group from the universal sulfate donor, 3′-phosphoadenosine 5′-phophosulfate (PAPS) to a tyrosine on the nascent protein [4]. The two TPSTs differ in their kinetics [5]–[7].

Among the tissues in which protein-tyrosine sulfation has been investigated is the retina. Multiple sulfated proteins are present in the retinas of different species and the transcripts for both *Tpst1 and Tpst2* are expressed [8]. Moreover, sulfated proteins are found in both the neural retina and the retinal pigment epithelium (RPE) [8], [9].

Complete lack of sulfation causes a drastic reduction in scotopic and photopic electroretinographic (ERG) responses and ultrastructural abnormalities in the rod outer segment (OS) characterized by membrane evulsions into the extracellular space, irregular disc membrane spacing, and expanded intradiscal space [10]. Surprisingly, rod photoreceptors continue to show normal function in single cell recordings in the absence of sulfation [10]. The complete absence of sulfation also affects establishment of neuronal circuits and may have effects on long-term synaptic maintenance [10].

**Table 1:** Primary antibodies and lectins used for tissue labeling.

Antigen	Host	Dilution	Source (catalog #; clone #)	Reference
Calbindin	Mouse	1∶300	Sigma Chemical Company, St. Louis, MO (C9848; clone CB955)	–
Calbindin	Rabbit	1∶1000–1∶5000	SWANT, Bellinzona, Switzerland (CB38)	–
Calretinin	Rabbit	1∶1000–1∶2000	Chemicon International, Temecula, CA (AB5054)	–
Chx-10	Sheep	1∶50	ExAlpha Biologicals, Inc., Watertown, MA (X1180P)	–
G_o_α	Mouse	1∶500–1∶1,000	Chemicon International, Temecula, CA (MAB3073; clone 2A)	[19]
Gγ13	Rabbit	1∶500	Dr. R. Margolskee Mount Sinai School of Medicine, New York, NY	[20], [21]
Glial Fibrillary Acidic Protein (GFAP)	Mouse	1∶500	Chemicon International, Temecula, CA (MAB360; Clone GA5)	[22]
Glutamic Acid Decarboxylase, 65 kDa (GAD-65)	Mouse	1∶500–1∶1000	Chemicon International, Temecula, CA (MAB351; clone GAD-6)	[23]
Glutamine synthetase	Mouse	1∶1000	Chemicon International, Temecula, CA (MAB302; clone GS6)	[24]
Islet-1	Mouse	1∶100–1∶200	Developmental Studies Hybridoma Bank (clone 39.4D5)	[18]
Microtubule AssociatedProtein 1 (MAP-1)	Mouse	1∶300–1∶500	Sigma Chemical Company, St. Louis, MO (M4278; clone HM-1)	[25]
Peanut Agglutinin (PNA)	–	1∶10–1∶20	Molecular Probes, Eugene, OR (L21409)	[26]
Protein Kinase C (PKC)	Mouse	1∶25–1∶100	BD Transduction Labs, San Jose, CA (610108; clone 3)	–
Protein Kinase C (PKC)	Rabbit	1∶1000–1∶2000	Sigma Chemical Company, St. Louis, MO (P-4334)	–
Sulfotyrosine	Human	1∶100–1∶1000	Dr. Kevin Moore, Oklahoma Medical Research Foundation, Oklahoma City OK. Antibody clone PSG-2	[9], [27]
Synapsin I	Rabbit	1∶500	Chemicon International, Temecula, CA (AB1543P)	[28]
Synaptic Vesicle Protein 2B (SV2B)	Rabbit	1∶500	Dr. Roger Janz, University of Texas Houston Medical School, Houston, TX	[29]
Synaptotagmin 2	Mouse	1∶200	Zebrafish International Resource Center, Eugene, OR (Clone ZNP-1)	[17], [30]
Syntaxin 3	Rabbit	1∶750–1∶1000	Novus Biologicals, Littleton, CO (NB100–1644)	[31]–[33]
Tyrosine hydroxylase (TH)	Mouse	1∶500	Chemicon International, Temecula, CA (MAB318; clone LNC1)	–
Vesicular glutamate transporter 1 (VGLUT1)	Guinea pig	1∶2500–1∶5000	Chemicon International, Temecula, CA (AB5905)	[34]
Vesicular glutamate transporter 3 (VGLUT3)	Guinea pig	1∶2500	Chemicon International, Temecula, CA (AB5421)	[35]
Wheat germ agglutinin (WGA)	–	1∶20	Molecular Probes, Eugene, OR (W11261)	[26]

To investigate the functional significance of protein-tyrosine sulfation by each of the two TPSTs, *Tpst1^−/−^* and *Tpst2^−/−^* mice were used. Here, we show that the absence of either TPST caused early functional deficits in the retina as assessed by ERG analysis. However, the ERG responses from *Tpst1^−/−^* retinas eventually attained amplitudes comparable to those observed in wildtype (*wt, Tpst1&2^+/+^*) mice by postnatal day (P) 50, then remained comparable to *wt* for the rest of the ages tested. The *Tpst2^−/−^* mice displayed early ERG deficits that, unlike *Tpst1^−/−^* mice, did not normalize with age. Although the complete lack of protein-tyrosine sulfation disrupts rod OS ultrastructure [10], the presence of either one of the two TPSTs was sufficient to maintain normal OS ultrastructure and retinal histology. Despite the functional impairments caused by the lack of either TPST, deficiency in either TPST-1 or TPST-2 did not result in any large-scale disruption of neuronal architecture or cell types, although somewhat diminished neuronal plexus in the inner retina were noted. These results suggest that the two TPSTs have some level of functional redundancy, although the distinct phenotypes of the *Tpst1^−/−^* and *Tpst2^−/−^* mice indicate that some retinal proteins must be selectively sulfated by either TPST-1 or -2.

**Figure 1:**
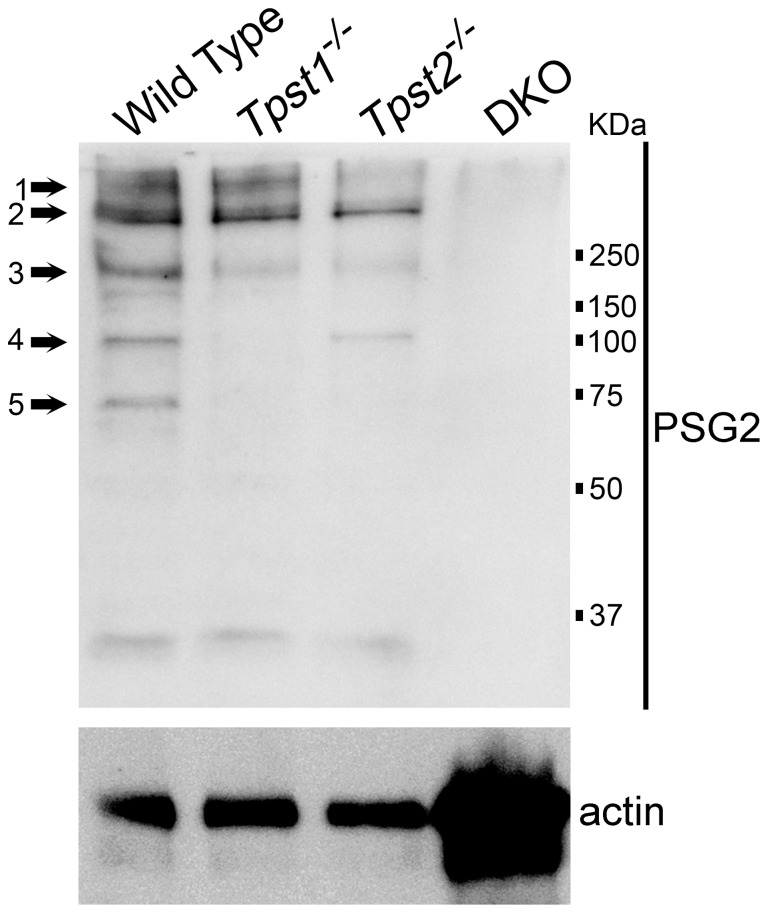
Immunoblot (Western) analysis of sulfated proteins in retinas of wildtype, *Tpst1^−/−^* and *Tpst2^−/−^* mice. Fifty micrograms of retinal protein extracts were loaded in each lane and 10% non-reducing SDS-PAGE was performed. Proteins were transferred to a nitrocellulose membrane and probed with the PSG2 antibody as described before [8]. The black arrows point to five bands of tyrosine sulfated proteins that show differential sensitivity to elimination of TPST-1 or TPST-2.

**Figure 2:**
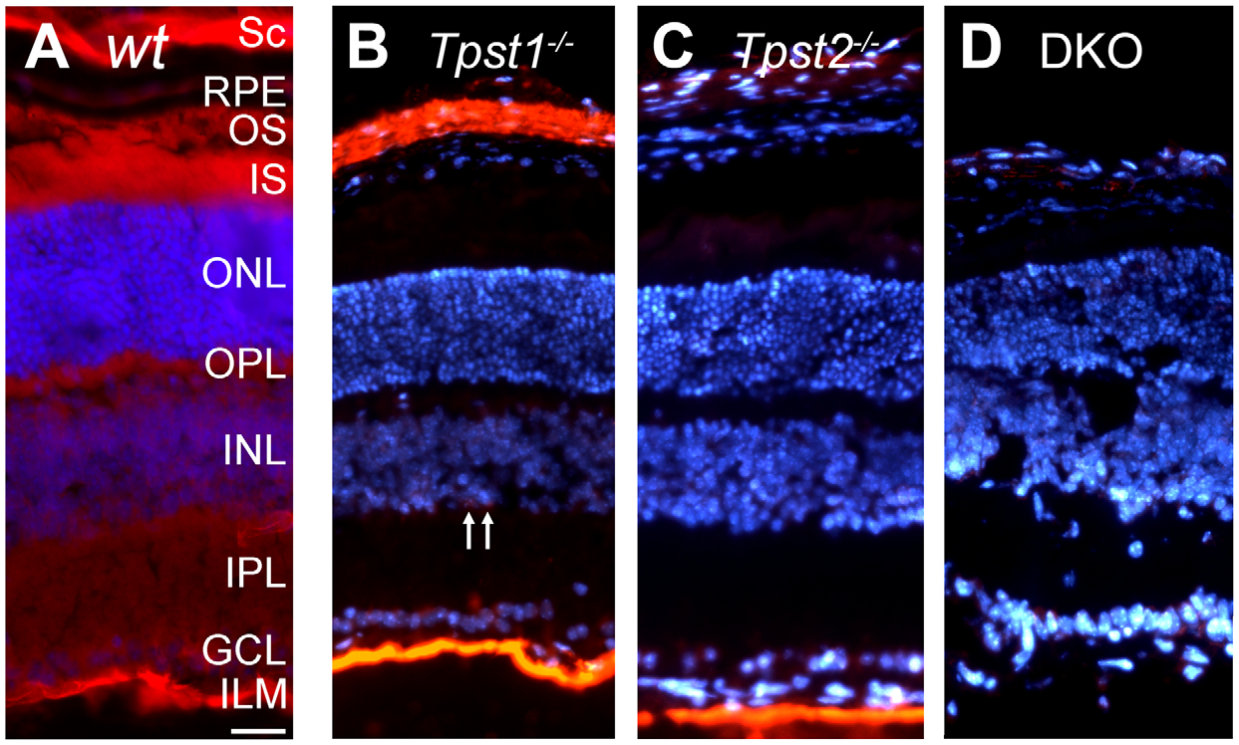
Immunohistochemical localization of sulfated proteins. Retinas from (**A**) wildtype (*wt*), (**B**) *Tpst1^−/−^,* (**C**) *Tpst2^−/−^* and (**D**) *Tpst* DKO mice were labeled using the PSG2 antibody. Labeling of *wt* retina showed signal in the sclera (SC), retinal pigment epithelium (RPE), photoreceptor outer segments (OS), inner segments (IS), outer nuclear layer (ONL), outer plexiform layer (OPL), inner nuclear layer (INL), inner plexiform layer (IPL) and ganglion cell layer (GCL). Arrows in panel B indicate cells in the proximal INL that retained sulfated proteins in absence of TPST-1. Retinas were from 30-day old *wt*, *Tpst1^−/−^* and *Tpst2^−/−^* mice while the DKO retina was from a 21-day old mouse. Scale bar  = 50 µm.

## Materials and Methods

### Animals

*Tpst1^−/−^* (Tpst1^tm1Klm^, MGI:2183366) and *Tpst2^−/−^* (Tpst2^tm1Klm^, MGI:3512111) were generated, characterized, housed, and fed as previously described [11]–[13]. This study was performed in strict accordance with the recommendations in the Guide for the Care and Use of Laboratory Animals of the National Institute of Health and the *Association for Research in Vision and Ophthalmology Resolution on the Use of Animals in Research*. The protocol was approved by the Institutional Animal Care and Use Committees at the University of Oklahoma Health Sciences Center (IACUC 09-024). In all animal experiments, every effort was made to minimize suffering.

### Electroretinography

Electroretinograms (ERGs) were recorded from the corneal surface of mice as described in detail previously [10].

**Figure 3:**
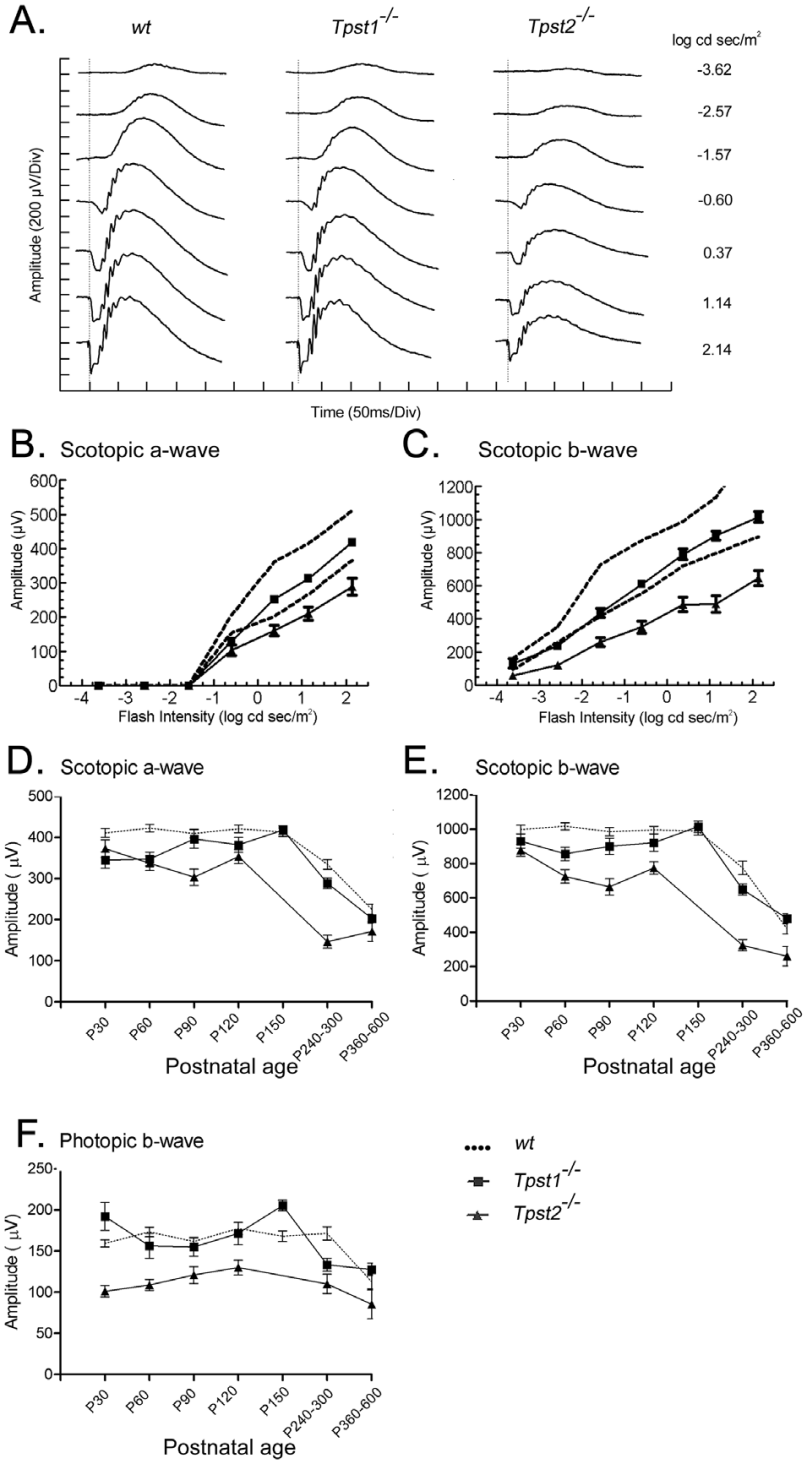
Electroretinographic responses from *Tpst1^−/−^* and *Tpst2^−/−^* retinas. **A.** Representative waveforms recorded at different light intensities from *wt*, *Tpst1^−/−^* or *Tpst2^−/−^* retinas under scotopic conditions. **B & C,** in each panel, the dashed line represents the 95% confidence interval for responses obtained from 3 150-day-old *wt* littermate controls. The a- and b-waves obtained from 3 150-day-old *Tpst1^−/−^* mice (squares) fell near the lower limit of this interval at lower light intensities, but were well within the range at higher light intensities. In contrast, a- and b-waves recorded from 3 123-day-old *Tpst2^−/−^* mice (triangles) were reduced in amplitude and fell outside the 95% confidence interval at all light intensities tested. Development of the (**D**) scotopic a-wave, (**E**) scotopic b-wave, and (**F**) photopic b-wave responses for *wt* (dotted line), *Tpst1^−/−^* (squares), and *Tpst2^−/−^* (triangles) mice. Note that all responses from *Tpst2^−/−^* mice were lower than those for *Tpst1^−/−^* and *wt* mice at all ages. Error bars represent standard error of the mean. The differences between *wt* and *Tpst2^−/−^* presented in **D** are statistically significant between P30 and P300 (P<0.05–0.001) while the differences between *wt* and *Tpst1^−/−^* are only statistically significant at P30 and P60 (P<0.05–0.001). The differences between *wt* and *Tpst2^−/−^* presented in **E** are statistically significant between P60 and P600 (P<0.01–0.001) while the differences between *wt* and *Tpst1^−/−^* are only statistically significant at P60 (P<0.05). The differences between *wt* and *Tpst2^−/−^* presented in **F** are statistically significant for all time points tested (P<0.05–0.001) while the differences between *wt* and *Tpst1^−/−^* are statistically insignificant for all time points tested. Number of animals tested in **D, E & F** are presented in Table 3.

### Light and Electron Microscopy

Methods used for tissue collection, processing for Spurr’s resin-embedment, microtomy and subsequent examination by light (LM) and electron microscopy (EM) were as previously described [10], [14].

### Immunohistochemistry and Lectin Cytochemistry

Immunohistochemistry (IHC) and lectin cytochemistry analyses were performed on tissue sections from frozen or paraffin-embedded eyes as described elsewhere [10]. Analyses were performed on retinas from 9–10 animals for each genotype at 1 to 2 months of age. The panel of well-characterized cell- and synapse-specific marker antibodies used in the present studies is described in **Table 1**.

### Immunoblotting (Western Analysis)

Immunoblotting was performed using the PSG2 antibody as described previously [15] and images were acquired using a Kodak image station (Carestream Molecular Imaging, Rochester, NY).

**Figure 4:**
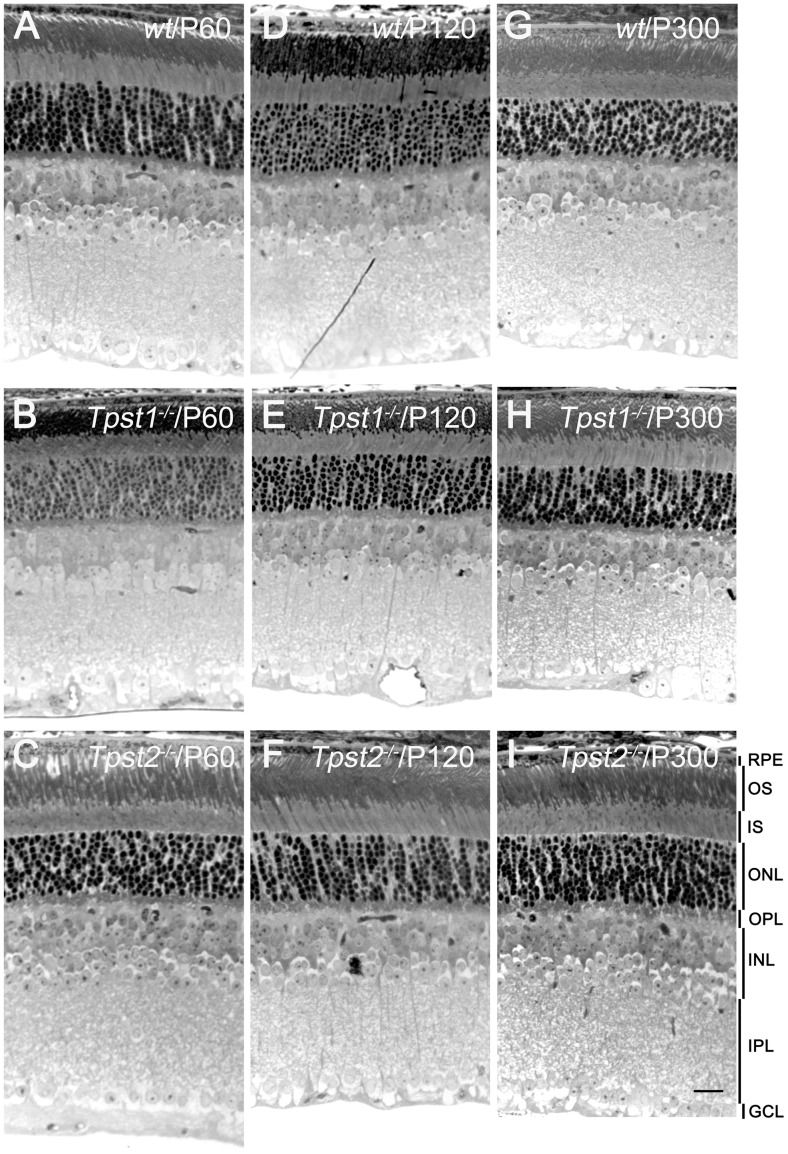
Histologic analysis in absence of either TPST-1 or TPST-2. **A, D &G,** histological appearance of *wt* retina at P60, P120 and P300, respectively; **B**, **E &** **H,** *Tpst1^−/−^* retina; **C**, **F**, & **I**, *Tpst2^−/−^* retina. Abbreviations as in Fig. 1. Scale Bar  = 50 µm.

### Statistical Analysis

Statistical significance was determined using ANOVA with Bonferroni *post hoc* multiple pairwise comparison tests (PRISM™; GraphPad® Software, San Diego, CA).

**Figure 5:**
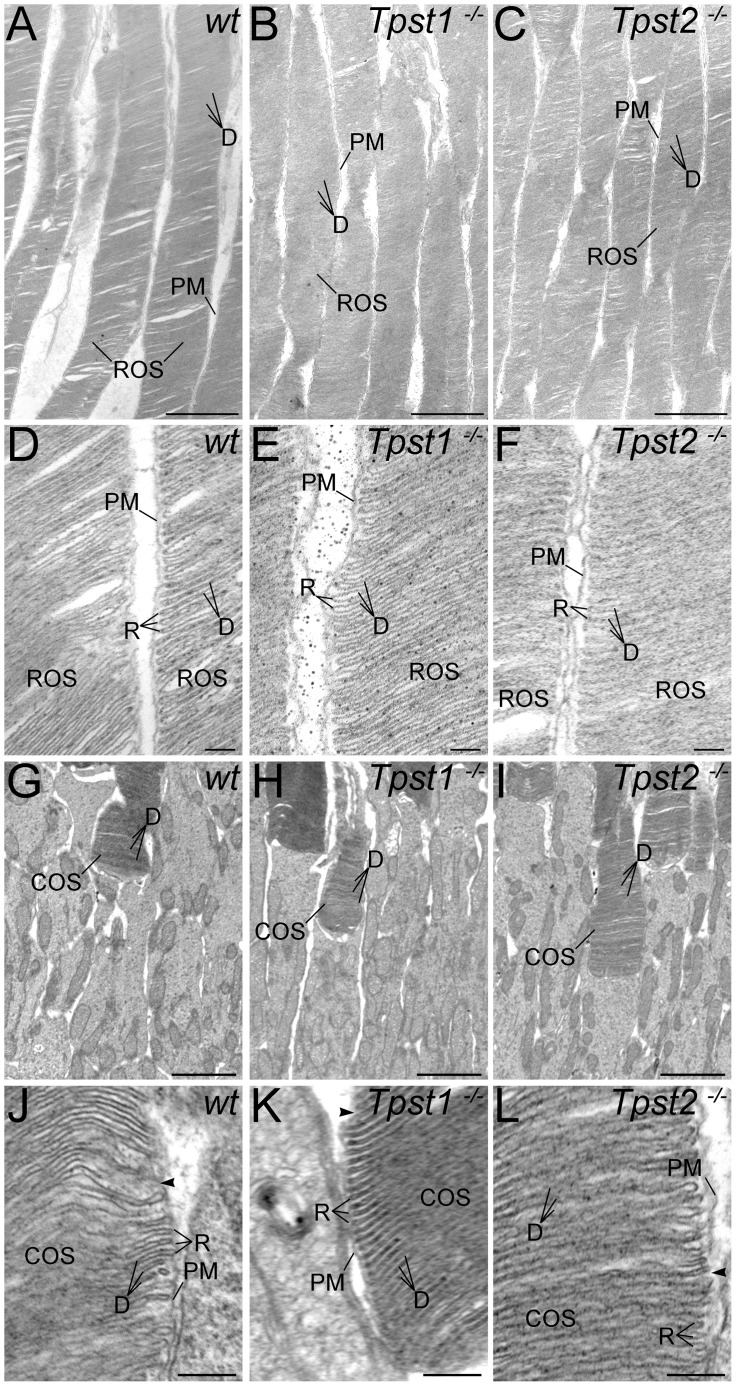
Knockout of TPST-1 or TPST-2 does not disrupt rod or cone outer segment ultrastructure. **A–C:** Rod outer segments (ROSs) in (**A**) *wt*, (**B**) *Tpst1^−/−^*, and (**C**) *Tpst2^−/−^* retinas. Wildtype, *Tpst1^−/−^*, and *Tpst2^−/−^* ROSs all show normal organization with closely stacked discs (**D**) surrounded by the plasma membrane (PM). **D–F:** ROS ultrastructure at higher magnification in (**D**) *wt*, (**E**) *Tpst1^−/−^*, and (**F**) *Tpst2^−/−^* retinas. ROSs in the retinas of mice of all three genotypes show normal flattened discs (**D**) with the typical hairpin organization of the disc rim (R). Discs are separate from the surrounding plasma membrane (PM) and have little intradiscal space. **G–I:** Cone outer segments (COSs) in (**G**) *wt*, (**H**) *Tpst1^−/−^*, and (**I**) *Tpst2^−/−^* retinas. COSs in the wildtype, *Tpst1^−/−^*, and *Tpst2^−/−^* retina all show the normal tapered shape, tightly packed, flattened discs, and positioning among the inner segments of neighboring rods. **J–L:** COS ultrastructure at higher magnification in (**J**) *wt*, (**K**) *Tpst1^−/−^*, and (**L**) *Tpst2^−/−^* retinas. COSs in the retinas of mice of all three genotypes show normal flattened discs, hairpin organization of the disc rim, and tight packing. The space between disks is continuous with the extracellular space (*arrowheads*). Scale bars  = 2 µm for A–C and G–I; 0.25 µm for D–F and J–L.

**Figure 6:**
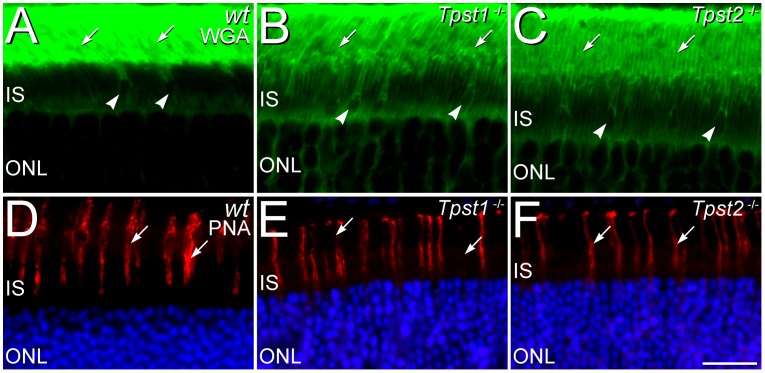
Knockout of TPST-1 or TPST-2 does not disrupt rod and cone-specific domains in the interphotoreceptor matrix (IPM). **A–C.** In the retinas of (**A**) *wt*, (**B**) *Tpst1^−/−^*, and (**C**) *Tpst2^−/−^* mice, the IPM surrounding rod outer segments (*arrows*) is labeled by wheat germ agglutinin (WGA; *green*). The IPM surrounding the inner and outer segments of cones (*arrowheads*) also shows labeling by WGA. **D–F:** Peanut agglutinin (PNA) specifically labels the IPM surrounding cones (*arrows*) in the retinas of (**D**) *wt*, (**E**) *Tpst1^−/−^*, and (**F**) *Tpst2^−/−^* mice, but, as appropriate, does not label the IPM surrounding rods. Photoreceptor nuclei in the outer nuclear layer (ONL) are counterstained with DAPI (blue) in panels D–F. IS, inner segment layer. Scale bars  = 20 µm for all panels.

## Results

### Distribution of Tyrosine-sulfated Proteins in Retinas of TPST-1 and TPST-2 Knockout Mice

Previous immunoblotting of extracts from *wt* retinas using the PSG2 anti-sulfotyrosine antibody showed that the retina contains several tyrosine-sulfated proteins with some of these proteins being specific to certain cell types [8]. To determine how the pattern observed on the immunoblots was affected by the elimination of either TPST, retinal extracts obtained from *Tpst1^−/−^* and *Tpst2^−/−^* mice were analyzed independently and compared to the pattern observed for *wt* retinal extracts. As shown in **Figure 1**, variations in 5 bands were observed on the immunoblots. In *Tpst1^−/−^* retinas, bands 2 and 3 were reduced, and bands 4 and 5 were absent. However, in *Tpst2^−/−^* retinas, bands 2, 3 and 4 were reduced in intensity, while bands 1 and 5 were absent. In DKO retinas, all bands were absent, confirming the specificity of PSG2 for sulfated proteins.

**Table 2:** Effects of *TPST-1* and *TPST-2* knockout on neuronal populations, morphology and synaptic protein expression.

Cell- or synapse-specific marker	Wildtype	*TPST-1* knockout	*TPST-2* knockout	*Tpst DKO* *
*Photoreceptors*				
PNA	Cone IPM and flat contacts with OFF-cone bipolar cells	Similar to wildtype	Similar to wildtype	Similar to wildtype
WGA	IPM surrounding rod outer segments	Similar to wildtype	Similar to wildtype	Similar to wildtype
VGLUT1	All rod and cone terminals	Similar to wildtype	Similar to wildtype	Similar to wildtype
SV2B	All rod and cone terminals	Similar to wildtype	Similar to wildtype	Similar to wildtype
Syntaxin 3	All rod and cone terminals	Present, but labeling may be reduced	Present, but labeling may be reduced	Present, but labeling may be reduced
*Bipolar cells*				
Synaptotagmin 2	Type 2 OFF-cone bipolar cells and Type 6 ON-cone bipolar cells	Normal Type 2 OFF-cone bipolar cells; expanded Type 6 ON-cone bipolar cell plexus in IPL	Normal Type 2 OFF-cone bipolar cells; expanded Type 6 ON-cone bipolar cell plexus in IPL	Normal Type 2 OFF-cone bipolar cells; expanded Type 6 ON-cone bipolar cell plexus in IPL
PKC-α	Rod bipolar cells and terminals	Similar to wildtype	Similar to wildtype	Similar to wildtype
Gγ13	All rod and ON-cone bipolar cell bodies and terminals	Similar to wildtype	Similar to wildtype	Labeling present in rod bipolar and ON-cone bipolar cells, but reduced.
Goα	All ON-type bipolar cell bodies. Other processes in IPL	Similar to wildtype	Similar to wildtype	Labeling present in rod bipolar and ON-cone bipolar cells, and in processes in IPL but reduced.
Chx-10	All bipolar cell nuclei	Similar to wildtype	Similar to wildtype	Similar to wildtype
Islet-1	All rod and ON-cone bipolar cell nuclei	Similar to wildtype	Similar to wildtype	Similar to wildtype
VGLUT1	All bipolar cell terminals	Similar to wildtype	Similar to wildtype	Similar to wildtype
SV2B	All bipolar cell terminals	Similar to wildtype	Similar to wildtype	Similar to wildtype
Syntaxin 3	All bipolar cell terminals	Similar to wildtype	Similar to wildtype	Similar to wildtype, but labeling may be reduced
*Horizontal cells*				
Calbindin	Horizontal cells	Similar to wildtype	Similar to wildtype, but plexus in OPL reduced	Similar to wildtype, but plexus in OPL reduced
*Amacrine cells*				
Calretinin	Starburst amacrine cells, TH2 amacrine cells (and ganglion cells)	Similar to wildtype	Similar to wildtype	Starburst and TH2 amacrine cell plexes severely reduced. (Ganglion cells similar to wildtype)
VGLUT3	Putative glutamatergic amacrine cells	Similar to wildtype	Process stratification similar to wildtype, but plexus reduced	VGLUT3 cells and plexus severely reduced
Tyrosine hydroxylase	Type 1 dopaminergic amacrine cells	Similar to wildtype	Process stratification similar to wildtype, but plexus reduced	Process stratification similar to wildtype, but plexus reduced
GAD-65	GABAergic amacrine cells and processes in IPL	Similar to wildtype	Process stratification similar to wildtype, but labeling reduced	Process stratification similar to wildtype, but labeling strongly reduced
*Ganglion cells*				
MAP-1	Ganglion cell dendrites and axons	Similar to wildtype	Similar to wildtype	Similar to wildtype
Calretinin	Ganglion cell bodies and axons (and starburst and TH2 amacrine cells)	Ganglion cell labeling similar to wildtype	Ganglion cell labeling similar to wildtype	Ganglion cell labeling similar to wildtype (amacrine cell labeling reduced)
*Glial cells*				
Glutamate synthetase	Müller cells and astrocytes	Similar to wildtype	Similar to wildtype	Similar to wildtype
GFAP	Astrocytes and Müller cell endfeet	Similar to wildtype	Similar to wildtype	Similar to wildtype

* *Tpst DKO* data from Sherry et al., 2010.

**Table 3:** Number of animals tested in Figure 3D, E & F.

Age/days	Wildtype	*Tpst1^−/−^*	*Tpst2^−/−^*
30	28	7	5
60	28	7	13
90	24	8	9
120	28	12	8
150	21	3	0
240–300	9	9	12
360–600	9	12	3

Immunohistochemical (IHC) analysis of sections of *wt* retina using the PSG2 antibody showed that tyrosine-sulfated proteins were present in all retinal layers and sclera (**Figure 2A**), consistent with previous findings [8]. To obtain clues as to the differential contributions of the two TPSTs to sulfation of retinal proteins, IHC analysis of tyrosine-sulfated proteins was performed on retinas of TPST-1 and TPST-2 knockout mice. In the *Tpst1^−/−^* retina, detection of tyrosine-sulfated proteins was observed in the ganglion cell layer (GCL), the inner limiting membrane (ILM), and a subset of cells in the proximal inner nuclear layer (INL) (**Figure 2B**) in addition to the expected strong immunolabeling of the sclera. Although the lack of TPST-2 (**Figure 2C**) did not affect immunolabeling in the ILM, it reduced labeling in the GCL, INL and sclera. Elimination of both enzymes [10] abolished anti-sulfotyrosine immunolabeling in all retinal layers (**Figure 2D**).

These results indicate that TPST-1 and -2 differentially sulfate some retinal proteins, while there may be some redundancy between the two enzymes for other substrates. Additionally, there could be multiple proteins in the ILM that are differentially sulfated by both TPSTs.

### Retinal Function and Structure in *Tpst1^−/−^* and *Tpst2^−/−^* Mice

We have previously shown that transcripts for both *Tpst1 and Tpst2* are expressed in human and mouse retina [8]. Both transcripts are expressed in the mouse retina as early as P1, although transcript levels fluctuate over the course of retinal development [10]. To determine the effect of the absence of either of the two TPSTs on retinal function, ERG analysis was performed. **Figure 3** presents dark-adapted ERGs obtained from representative *wt*, *Tpst1^−/−^* and *Tpst2^−/−^* mice to flash stimuli that span an intensity range of several log units, including the range from purely rod-driven (scotopic) responses, to mixed rod- and cone-driven responses, to purely cone-driven (photopic) responses (**Figure 3A**). At the lowest flash intensity, the response of *wt* mice was dominated by the positive-going b-wave, reflecting the activity of rod bipolar cells and other cells in the inner retina. With increasing flash intensities, the b-wave was preceded by the negative-going a-wave, representing the mass response of the rod photoreceptors. In *Tpst1^−/−^* mice, the a-wave was reduced in amplitude compared to *wt* at low stimulus intensities, but fell within the normal range for higher light intensities (**Figure 3B&C**). In contrast, in *Tpst2^−/−^* mice, both the a- and b-wave were reduced in amplitude at all stimulus intensities. While the average intensity-response functions for the a- and b-waves in *Tpst1^−/−^* mice fell within the 95% confidence interval defined in *wt* mice, the a- and b-wave amplitudes for *Tpst2^−/−^* fell outside the 95% confidence interval (dashed lines, **Figures 3B&C**).

Developmentally, the scotopic a- and b-wave amplitudes of *Tpst1* ^−/−^ mice started significantly below those observed in age-matched *wt* mice, but increased to *wt* levels by P90 (**Figure 3D&E**). In contrast, the amplitude of the photopic b-wave from *Tpst1^−/−^* retinas was comparable to *wt* retinas even at early ages (**Figure 3F**). *Tpst2* ^−/−^ mice showed a more severe functional impairment, with the scotopic a- and b-wave amplitude starting smaller than the *wt* level and never achieving *wt* levels until after P360 (**Figure 3D, E&F**), although there was a slight, but significant (P≤0.0163), improvement in amplitudes between P90 and P120. The insignificant difference in ERG responses between *wt* and *Tpst2^−/−^* mice after P360 is mostly due to reduced responses in aging *wt* mice. However, it is possible that lack of TPST-2 may provide some protective effects against age-related changes. There also was a small increase in photopic b-wave amplitudes between P30 and P120 for *Tpst2^−/−^* mice. Nevertheless, the responses never reached the amplitudes observed for *Tpst1^−/−^* or *wt* mice. The decline in photopic ERG responses for both *Tpst1^−/−^* and *Tpst2^−/−^* mice past P150 paralleled that exhibited by *wt* mice. Together, these data suggest that TPST-2 is more important to retinal function than is TPST-1.

The reduction in both scotopic and photopic function in *Tpst2^−/−^* retinas could potentially arise from degenerative changes in rods and cones, in second-order neurons, or both. Alternatively, reduced function might reflect subtle functional or anatomical changes independent of degeneration. However, examination of *wt*, *Tpst1^−/−^*, and *Tpst2^−/−^* retinas at the light microscopic levels revealed no obvious histologic anomalies (**Figure 4**).

Although double knockout (DKO) of TPST-1 and TPST-2 causes a profound disruption of rod OS (ROS) [10], ROS ultrastructure was unaffected by knockout of either *Tpst1^−/−^* or *Tpst2^−/−^* and was comparable to that of *wt* ROS (**Figure 5**). Cone OS (COS) ultrastructure also was unaffected in either *Tpst1^−/−^* and *Tpst2^−/−^* retinas, consistent with the normal COS structure previously observed in the *Tpst* DKO retina [10]. Similarly, the rod- and cone-specific domains in the interphotoreceptor matrix (IPM) around the outer segments of rods and cones also appeared unaffected by elimination of either TPST-1 or TPST-2, as assessed by lectin cytochemistry (**Figure 6**).

### Single Knockout of TPST-1 or TPST-2 has Minimal Effects on Retinal Cell Populations, Morphology, or Organization of Retinal Neurons and Synapses

To test whether the deficits in retinal function in *Tpst1^−/−^* and *Tpst2^−/−^* mice might arise from aberrant neuronal morphology or synaptic organization, we examined the expression and localization of a number of cell- and synapse-specific markers in the retinas of *Tpst1^−/−^* and *Tpst2^−/−^* mice (**Table 1**). All populations of retinal neurons examined were present in the *Tpst1^−/−^* and *Tpst2^−/−^* retinas and showed appropriate cell-specific morphology (summarized in **Table 2** **; data shown in Figures S1 and S2**). Although all cell types were present in the *Tpst1^−/−^* and *Tpst2^−/−^* retina, in some cases the extent of their plexes were slightly diminished compared to *wt*, with knockout of TPST-2 having a slightly stronger effect than knockout of TPST-1. The Type 6 ON-cone bipolar cell was an exception to this pattern as it showed a small expansion of its narrow plexus in the inner plexiform layer (IPL) in both *Tpst1^−/−^* and *Tpst2^−/−^* retinas (**Figure S2**), consistent with findings in the *Tpst* DKO retina [10]. Although elimination of either TPST-1 or TPST-2 affected retinal function, the structural organization of retinal circuits, which reflects their function, in the *Tpst1^−/−^* and *Tpst2^−/−^* retina was unaffected (**Table 2** **; Figures S1&S2**). Retinal neurons projected to only appropriate regions of the outer plexiform layer (OPL) and inner plexiform layer (IPL), with the IPL retaining its normal ON-OFF segregation. Similarly, knockout of either TPST-1 or TPST-2 did not affect synapse-specific expression of presynaptic proteins associated with vesicular neurotransmitter release or the expression of G-protein subunits associated with transmission from photoreceptors to rod or ON-type cone bipolar cells (**Table 2**). Müller glial cells, the principal glial cell of the retina, showed normal morphology with no elevated expression of glial fibrillary acid protein (GFAP) (**Figure S3**), a sensitive indicator of retinal stress or degeneration [16]. Thus, the deletion of TPST-1 or -2 did not disrupt cell populations, cell-specific projection patterns or the structural or functional organization of the retina.

Electron microscopy confirmed that all types of retinal neurons established ultrastructurally appropriate synapses in the *Tpst1^−/−^* and *Tpst2^−/−^* retina (**Figure S4**). The synaptic terminals of rods and cones showed normal synaptic ultrastructure, with synaptic ribbon complexes showing the classic post-synaptic triadic organization of horizontal cell and bipolar cell processes aligned with the synaptic ribbon apparatus in the presynaptic photoreceptor terminal. Flat contacts between cones and the dendrites of OFF-cone bipolar cells were also present. Bipolar cell synapses showed the normal diadic arrangement of two post-synaptic processes apposed to a small presynaptic ribbon in the bipolar cell terminal, and amacrine cells established conventional vesicular synapses.

Altogether, these results indicate that retinal neurons differentiate normally in the absence of either TPST-1 or TPST-2. Furthermore, these findings suggest that the functional deficits observed in the ERGs recorded from the *Tpst1^−/−^* and *Tpst2^−/−^* retinas do not originate from large-scale defects in retinal cell populations, the loss of basic cellular and synaptic organization or defects in the ability to establish synapses.

## Discussion

In a previous study, we showed that the complete absence of protein tyrosine sulfation due to knockout of both TPST-1 and TPST-2 severely disrupted the integrity of rod OS, resulting in abnormal disc morphology and protrusion of rod outer segment membranes into the subretinal space, while leaving cone outer segment ultrastructure intact [10]. To investigate the specific roles of TPST-1 and TPST-2 in sulfating retinal proteins and retinal development and homeostasis, mice with deletion of either TPST-1 or TPST-2 were used.

*Tpst1^−/−^* mice exhibited a transient developmental delay of ERG responses that reached normal levels by P90. Normalization of the ERG suggests that redundancy exists in the functions of TPST-1 and TPST-2, whereby TPST-2 may compensate to some degree for the absence of TPST-1 during retinal development. This is supported by the fact that in absence of both TPSTs, there is a clear functional and structural phenotype [10]. Since the functional recovery in the absence of TPST-1 is slow, it is likely that the TPST-2 exhibits differential affinity for TPST-1 substrates. However, the differential effects on protein sulfation observed by western blots and the differential distribution of sulfated proteins in retinas of *Tpst1^−/−^* and *Tpst2^−/−^* mice suggests that the two enzymes are not fully redundant in their functions.

*Tpst2^−/−^* mice displayed reduced scotopic and photopic ERG responses at P30; however, these functional deficits showed little or no improvement with age, in contrast to the ERG deficits of *Tpst1^−/−^* mice. The persistence of the functional defects in *Tpst2^−/−^* mice suggests that TPST-1 cannot fully compensate adequately for the absence of TPST-2 and vice versa. This also implies that some sulfated retinal proteins can only be sulfated by one of the two enzymes and supports the notion that these two enzymes are only partially redundant.

A key finding of these studies is that single knockout of either TPST-1 or TPST-2 results in a primarily functional phenotype, with little effect on retinal structure. Single knockout of either TPST-1 or TPST-2 had no effect on rod or cone outer segment ultrastructure. Similarly, the rod- and cone-specific domains within the IPM, as visualized by lectin cytochemistry, appeared normal in single knockout retinas. Although some very subtle structural changes in retinal neurons and their synaptic plexus were noted in the retinas of mice with single knockout of either TPST-1 or TPST-2, the resulting deficits in protein tyrosine sulfation did not disrupt retinal cell populations, neuronal morphology, organization, or their ability to form ultrastructurally appropriate synapses to any substantial degree. The principal effect on retinal structure and organization was a subtle, generalized reduction in the extent of neuronal plexus, which was more pronounced in the *Tpst2^−/−^* than in the *Tpst1^−/−^* retina, but less extensive than the reduction of neuronal plexus observed in the retinas of *Tpst* DKO mice [10].

One intriguing finding from our prior studies on the *Tpst* DKO mouse was the ability of rod photoreceptor cells to generate normal responses to light when determined by suction electrode recordings of isolated rod photoreceptor cells; in contrast, the global rod response (as recorded by the scotopic ERG a-wave) was significantly depressed and rod OSs showed severe disorganization at the ultrastructural level *in vivo* [10]. By extension, it is reasonable to suggest that single rod photoreceptors from *Tpst1^−/−^* or *Tpst2^−/−^* retinas should exhibit normal responses in suction electrode recordings. Taken together, the differences in rod responses *in vivo* and *in vitro* suggest that sulfation of protein(s) in the IPM is a critical determinant of the generation of normal rod responses *in vivo*.

The ERG b-wave deficits present in both *Tpst1^−/−^* and *Tpst2^−/−^* mice indicate that sulfation deficits impair synaptic transmission by photoreceptors to the inner retina and/or processing within the inner retina. Our studies indicate that the absence of sulfation by TPST-1 or TPST-2 does not cause large aberrations in retinal cell populations, morphology or neuronal architecture, although the neuronal plexus in the retina of sulfation-deficient mice tended to be diminished slightly compared to *wt* mice. The slightly more pronounced reduction of neuronal plexus in *Tpst2^−/−^* mice correlated with the severity of the ERG deficits, consistent with the more obvious reduction of neuronal plexus and retinal function seen in the *Tpst* DKO mouse [10]. Our ultrastructural studies confirmed that the functional deficits observed in the ERG did not arise from the failure of synapse formation *per se*, although the possibility remains that sulfation deficits could affect the numbers or specificity of synaptic connections. These findings support the notion that TPST-1 and TPST-2 have somewhat redundant functions and can compensate for the loss of a single isoform.

The current studies, together with our previous studies of the *Tpst* DKO mouse [10], indicate that sulfation by both TPST-1 and TPST-2 can affect retinal circuitry, although the effects of sulfation deficiency manifest themselves more clearly at the functional level than at the anatomical or ultrastructural levels. The mechanisms by which sulfation might regulate neuronal development and synaptic transmission are not known currently. Given that sulfation is a common modification of secreted proteins, one attractive possibility is that sulfated proteins secreted into the extracellular space or incorporated into the soluble and insoluble fractions of the extracellular matrix [8], provide signals controlling synaptic maturation and/or function.

The retinal phenotypes in *Tpst1^−/−^* or *Tpst2^−/−^* mice described above could result from global lack of tyrosine sulfation rather than lack of sulfation of retinal proteins. However, since the developmental functional deficit in the *Tpst2^−/−^* retina never recovers, it is most likely that these phenotypes are due to lack of sulfation of specific retinal proteins. Nevertheless, the ultimate answer will require tissue and/or cell type-specific conditional knockout experiments.

In summary, the data presented here in conjunction with our previous findings [10] demonstrate that protein-tyrosine sulfation is a key determinant in the development and maintenance of retinal function with more subtle effects on retinal structure. Studies are underway to identify tyrosine sulfated retinal proteins in order to better understand the role of sulfation in retinal function and to delineate how the lack of sulfation affects the functions of specific retinal proteins.

## Supporting Information

Figure S1 **Lack of TPST-1 or TPST-2 does not induce any large scale disruption of retinal horizontal, amacrine or ganglion cells. A–C:** Horizontal cells labeled for calbindin in the *wt*, *Tpst1^−/−^* and *Tpst2^−/−^* retina show normal placement in the inner nuclear layer (INL) and project normally to the outer plexiform layer (OPL), although the extent of their plexus in the *Tpst2^−/−^* retina is slightly reduced. **D–F:** Starburst, TH2, and ganglion cell populations label for calretinin and form three distinct projections (1,2,3) in the inner plexiform layer (IPL) as appropriate in the *wt*, *Tpst1^−/−^* and *Tpst2^−/−^* retina. **G–I:** GABAergic amacrine cells and their processes in the IPL of the *wt, Tpst1^−/−^* and *Tpst2^−/−^* retina, show normal labeling for the 65 kDa form of glutamic acid decarboxylase (GAD-65). Lamination of GABAergic amacrine cell processes in the IPL is also normal (*arrowheads*). **J–L:** A small population of amacrine cells (*arrowheads*) shows appropriate labeling for vesicular glutamate transporter 3 (VGLUT3) in the *wt*, *Tpst1^−/−^* and *Tpst2^−/−^* retina. **M–O:** Ganglion cells, their axons (Ax) and their dendrites in the *wt*, *Tpst1^−/−^* and *Tpst2^−/−^* retina show labeling for microtubule-associated protein 1 (MAP-1) as appropriate. Abbreviations as in Fig. 1. Scale bars  = 50 µm.
(TIF)

Figure S2 **Elimination of TPST-1 or TPST-2 does not induce any large scale disruption of retinal bipolar cells. (A–D)** *wt* retina; **(E–H)** *Tpst1^−/−^*; **(I–L)** *Tpst2^−/−^* retina. The projections of rod bipolar cells (labeled for protein kinase C (PKC), *green*; panels A, E, I) and Type 2 and Type 6 Cone bipolar cells labeled for synaptotagmin 2 (*blue*; panels C, G, K) show appropriate morphology and project appropriately to the ON and OFF sublayers of the inner plexiform layer (IPL). The Type 6 cone bipolar cell plexus in the ON sublayer of the IPL (*arrowheads*) is slightly expanded in TPST-1 and TPST-2 knockout retinas. The terminals of photoreceptors in the outer plexiform layer (OPL) and bipolar cell terminals in the inner plexiform layer (IPL) express vesicular glutamate transporter 1 (VGLUT1, *red*; panels B,F,J) as appropriate. Labeling of blood vessels (bv) in panels C,G,K is non-specific. Abbreviations as in Fig. 1. Scale bars  = 50 µm.
(TIF)

Figure S3 **Absence of TPST-1 or TPST-2 does not disrupt Müller glial cells. A–C:** Müller cells in *wt*, *Tpst1^−/−^* and *Tpst2^−/−^* retina show normal morphology and express glutamine synthetase (GS) as appropriate. Labeling of blood vessels (bv) is non-specific. **D–F:** Müller cells in the *wt*, *Tpst1^−/−^* and *Tpst2^−/−^* retina are not reactive and show normal localization of glial fibrillary acidic protein (GFAP, *red*) to the end feet (*arrowheads*) along the inner retinal margin. Nuclei are labeled with DAPI (*white*) to illustrate retinal layering. Labeling of blood vessels (bv) is non-specific. Abbreviations as in Fig. 1. Scale bars  = 50 µm.
(TIF)

Figure S4 **Development of normal synaptic ultrastructure in** ***Tpst1^−/−^*** **and** ***Tpst2^−/−^*** **retinas. A–C:** Rod terminals in (**A**) *wt*, (**B**) *Tpst1^−/−^*, and (**C**) *Tpst2^−/−^* retinas show normal ultrastructural organization. Post-synaptic triads comprised of horizontal cell processes (h) in the lateral position and a rod bipolar cell dendrite (b) in the central position are arranged around a synaptic ribbon (r) attached to the presynaptic membrane. **D–F:** Cone terminals in (**D**) *wt*, (**E**) *Tpst1^−/−^* and (**F**) *Tpst2^−/−^* retinas show normal ultrastructural organization. Cones from mice of all three genotypes made multiple synaptic ribbon complexes arranged around a synaptic ribbon attached to the plasma membrane of the cone terminal with the normal triad of two horizontal cell processes and a bipolar cell dendrite. In addition, cones also made flat contacts (fc, and *inset* in panel E) with bipolar cell dendrites as appropriate. **G–I:** Bipolar cell terminals in (**G**) *wt*, (**H**) *Tpst1^−/−^*, and **(I)** *Tpst2^−/−^* retinas show normal ultrastructural organization. Bipolar cells from mice of all three genotypes made normal synaptic complexes arranged around a short synaptic ribbon attached to the plasma membrane of the bipolar terminal with a dyad of post-synaptic processes (post 1 and post 2) arising from amacrine and ganglion cells. **J–L:** Conventional synapses made by amacrine cells in (**J**) *wt*, (**K**) *Tpst1^−/−^*, and (**L**) *Tpst2^−/−^* retinas show normal ultrastructure with synaptic vesicles (SV) presynaptically, a widened synaptic cleft, and densification of the pre- and post-synaptic membranes. Scale bars  = 0.5 µm for A–F; 0.2 µm for G–L.
(TIF)
