# Supplementary figures and images for: Differential Developmental Deficits in Retinal Function in the Absence of either Protein Tyrosine Sulfotransferase-1 or -2
Source: PLoS One. 2012 Jun 22;7(6):e39702. doi: 10.1371/journal.pone.0039702 (PMC3382163; doi:10.1371/journal.pone.0039702)

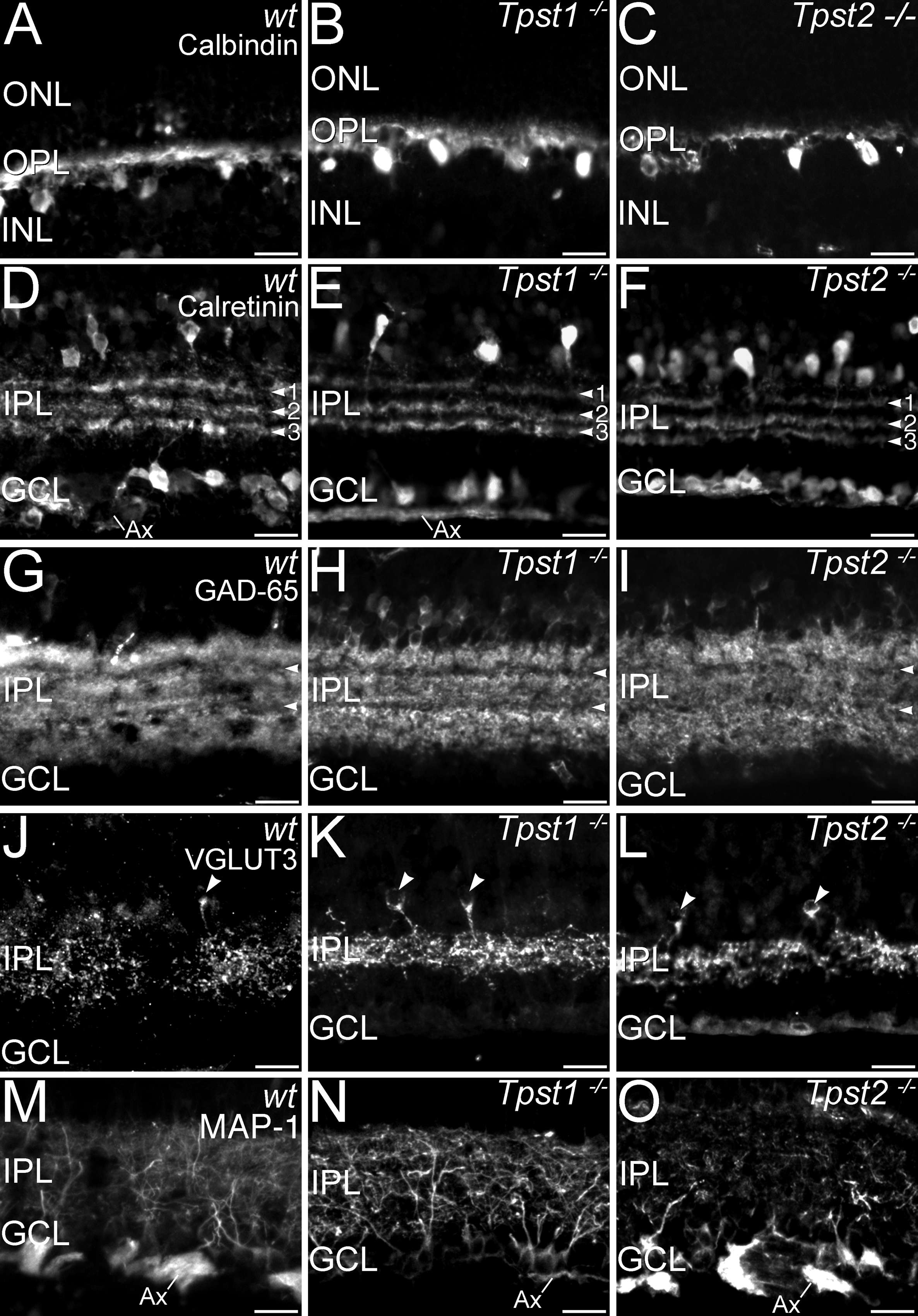

Supplement: Figure S1 — Lack of TPST-1 or TPST-2 does not induce any large scale disruption of retinal horizontal, amacrine or ganglion cells. A–C: Horizontal cells labeled for calbindin in the wt, Tpst1−/− and Tpst2−/− retina show normal placement in the inner nuclear layer (INL) and project normally to the outer plexiform layer (OPL), although the extent of their plexus in the Tpst2−/− retina is slightly reduced. D–F: Starburst, TH2, and ganglion cell populations label for calretinin and form three distinct projections (1,2,3) in the inner plexiform layer (IPL) as appropriate in the wt, Tpst1−/− and Tpst2−/− retina. G–I: GABAergic amacrine cells and their processes in the IPL of the wt, Tpst1−/− and Tpst2−/− retina, show normal labeling for the 65 kDa form of glutamic acid decarboxylase (GAD-65). Lamination of GABAergic amacrine cell processes in the IPL is also normal (arrowheads). J–L: A small population of amacrine cells (arrowheads) shows appropriate labeling for vesicular glutamate transporter 3 (VGLUT3) in the wt, Tpst1−/− and Tpst2−/− retina. M–O: Ganglion cells, their axons (Ax) and their dendrites in the wt, Tpst1−/− and Tpst2−/− retina show labeling for microtubule-associated protein 1 (MAP-1) as appropriate. Abbreviations as in Fig. 1. Scale bars = 50 µm. (TIF) [file pone.0039702.s001.tif]

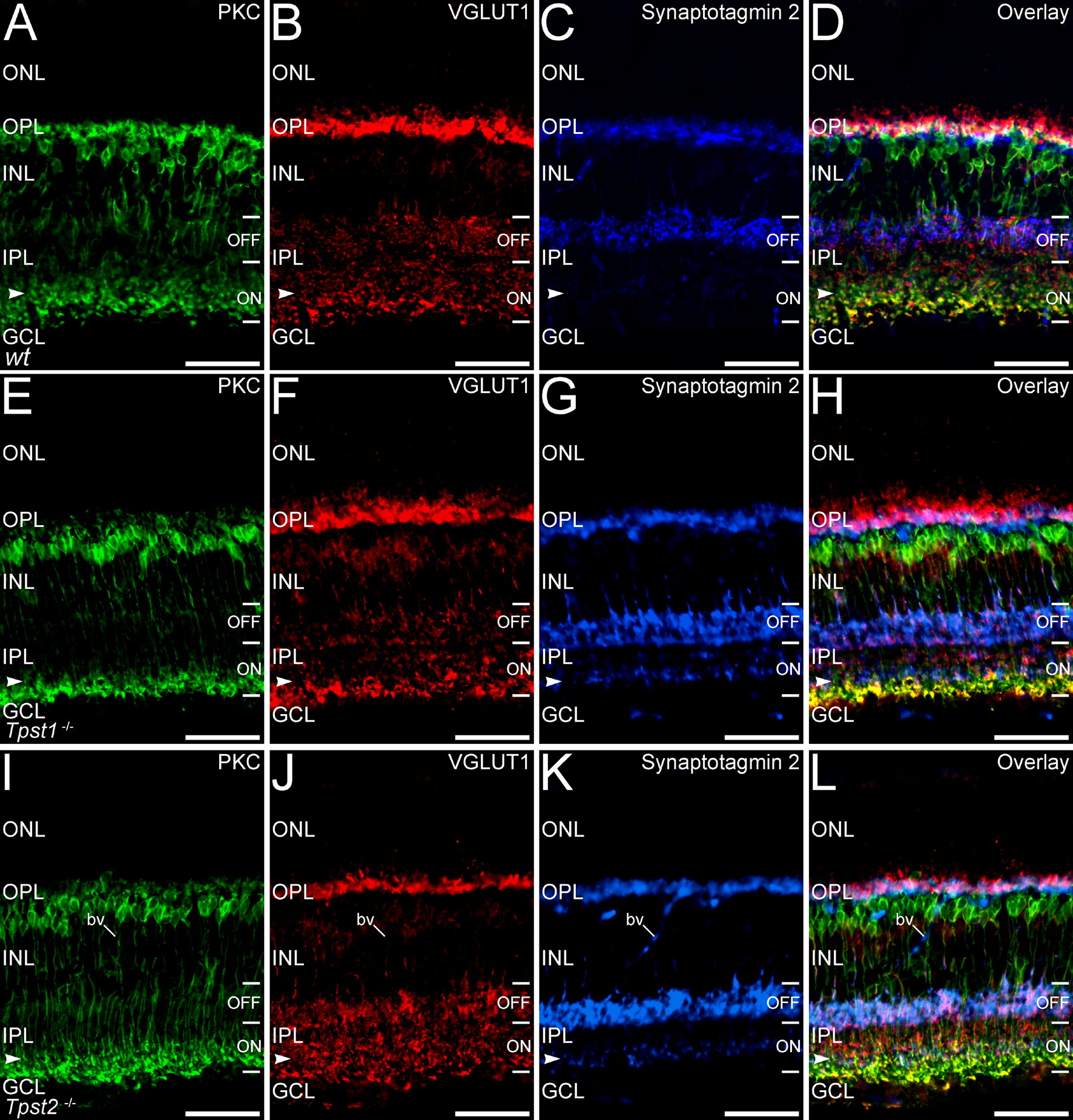

Supplement: Figure S2 — Elimination of TPST-1 or TPST-2 does not induce any large scale disruption of retinal bipolar cells. (A–D) wt retina; (E–H) Tpst1−/−; (I–L) Tpst2−/− retina. The projections of rod bipolar cells (labeled for protein kinase C (PKC), green; panels A, E, I) and Type 2 and Type 6 Cone bipolar cells labeled for synaptotagmin 2 (blue; panels C, G, K) show appropriate morphology and project appropriately to the ON and OFF sublayers of the inner plexiform layer (IPL). The Type 6 cone bipolar cell plexus in the ON sublayer of the IPL (arrowheads) is slightly expanded in TPST-1 and TPST-2 knockout retinas. The terminals of photoreceptors in the outer plexiform layer (OPL) and bipolar cell terminals in the inner plexiform layer (IPL) express vesicular glutamate transporter 1 (VGLUT1, red; panels B,F,J) as appropriate. Labeling of blood vessels (bv) in panels C,G,K is non-specific. Abbreviations as in Fig. 1. Scale bars = 50 µm. (TIF) [file pone.0039702.s002.tif]

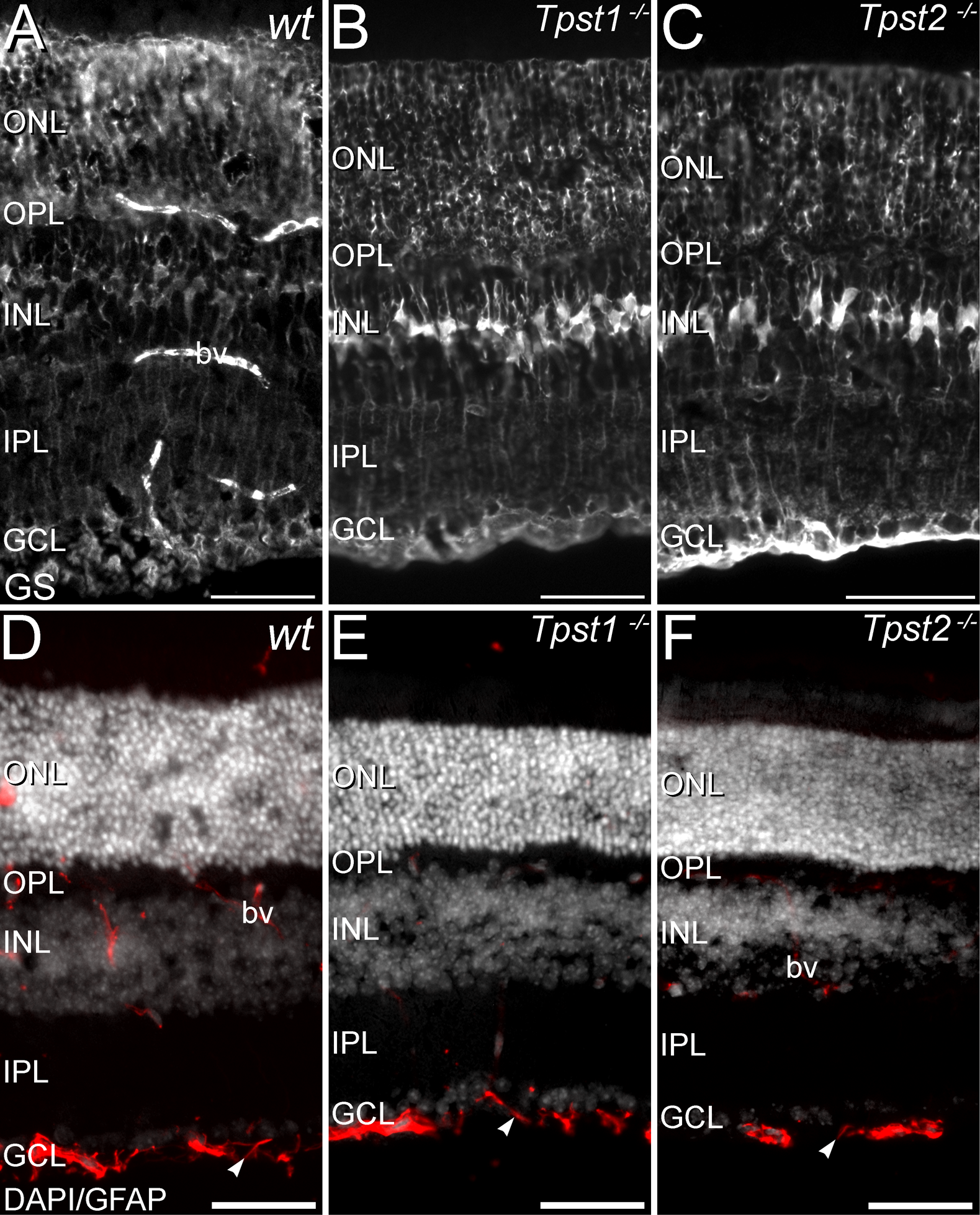

Supplement: Figure S3 — Absence of TPST-1 or TPST-2 does not disrupt Müller glial cells. A–C: Müller cells in wt, Tpst1−/− and Tpst2−/− retina show normal morphology and express glutamine synthetase (GS) as appropriate. Labeling of blood vessels (bv) is non-specific. D–F: Müller cells in the wt, Tpst1−/− and Tpst2−/− retina are not reactive and show normal localization of glial fibrillary acidic protein (GFAP, red) to the end feet (arrowheads) along the inner retinal margin. Nuclei are labeled with DAPI (white) to illustrate retinal layering. Labeling of blood vessels (bv) is non-specific. Abbreviations as in Fig. 1. Scale bars = 50 µm. (TIF) [file pone.0039702.s003.tif]

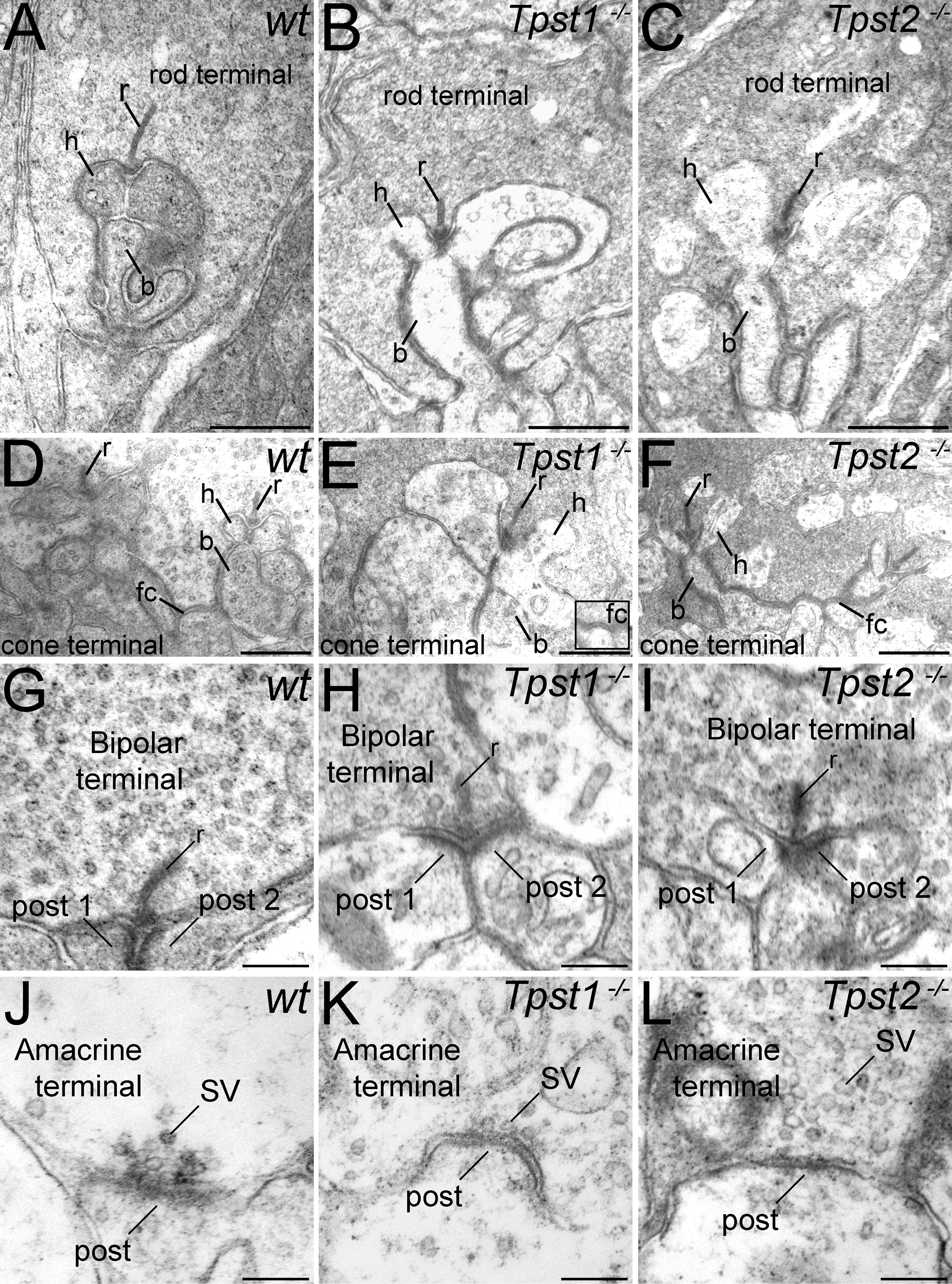

Supplement: Figure S4 — Development of normal synaptic ultrastructure in Tpst1−/− and Tpst2−/− retinas. A–C: Rod terminals in (A) wt, (B) Tpst1−/−, and (C) Tpst2−/− retinas show normal ultrastructural organization. Post-synaptic triads comprised of horizontal cell processes (h) in the lateral position and a rod bipolar cell dendrite (b) in the central position are arranged around a synaptic ribbon (r) attached to the presynaptic membrane. D–F: Cone terminals in (D) wt, (E) Tpst1−/− and (F) Tpst2−/− retinas show normal ultrastructural organization. Cones from mice of all three genotypes made multiple synaptic ribbon complexes arranged around a synaptic ribbon attached to the plasma membrane of the cone terminal with the normal triad of two horizontal cell processes and a bipolar cell dendrite. In addition, cones also made flat contacts (fc, and inset in panel E) with bipolar cell dendrites as appropriate. G–I: Bipolar cell terminals in (G) wt, (H) Tpst1−/−, and (I) Tpst2−/− retinas show normal ultrastructural organization. Bipolar cells from mice of all three genotypes made normal synaptic complexes arranged around a short synaptic ribbon attached to the plasma membrane of the bipolar terminal with a dyad of post-synaptic processes (post 1 and post 2) arising from amacrine and ganglion cells. J–L: Conventional synapses made by amacrine cells in (J) wt, (K) Tpst1−/−, and (L) Tpst2−/− retinas show normal ultrastructure with synaptic vesicles (SV) presynaptically, a widened synaptic cleft, and densification of the pre- and post-synaptic membranes. Scale bars = 0.5 µm for A–F; 0.2 µm for G–L. (TIF) [file pone.0039702.s004.tif]
